# The Health System in Costa Rica: Focus on the Management of Diabetes Mellitus

**DOI:** 10.7759/cureus.40084

**Published:** 2023-06-07

**Authors:** Chih H Chen Ku, Daniel Chen-Sandi

**Affiliations:** 1 Endocrinology, Clinica Los Yoses, San José, CRI

**Keywords:** treatment of diabetes mellitus, healthcare costs of diabetes, antidiabetic drugs, costa rica, diabetes mellitus management, global healthcare systems

## Abstract

Costa Rica’s health system was established in 1941 by the president at the time, Rafael Angel Calderon Guardia. Since then, the public health system has expanded, and a private system was also introduced. Diabetes management differs greatly between both systems, as well as the medications available.

Publicly, the system faces many challenges when treating diabetes; including a limited range and selection of medications, as well as a blatant lack of support (nutritional, physical, and psychological). Privately, the costs adjacent to a diabetes diagnosis represent an unbearable burden to some patients, with medications such as a weekly dose of 1.0 mg of semaglutide representing approximately 47.5% of Costa Rica’s minimum wage.

Despite its flaws, both systems provide the Costa Rican population with options for treatment. The Caja Costarricense de Seguro Social covers around 90% of the population, which puts Costa Rica on par with developed countries.

## Editorial

Introduction

Costa Rica’s health system first became centralized and widely available for the public in 1941, thanks to the president at the time Rafael Ángel Calderón Guardia, who founded the Caja Costarricense de Seguro Social (abbreviated to CCSS) [1]. Since then, Costa Rican citizens have had access to free healthcare in any public hospital or regional clinic. The public system is funded by both citizens and the government. In Costa Rica, all employers must pay a quota reduced from the worker’s salary to the CCSS in order to ensure workers are treated. Additionally, the patron must contribute to the CCSS as well as the government. Even before the existence of CCSS, private healthcare has been available and patients must pay in order to receive medical services.

Costa Rica has shown recent increasing trends in the prevalence of obesity and diabetes mellitus, similar to those presented in Europe and the United States [2], with 12.8% [3] of the adult population suffering from diabetes and 68.5% suffering from excess body weight (obesity and overweight combined) [4].

The minimum wage for Costa Rica for 2023 is 11,738.83 colones per day, equivalent to 352,164.9 colones per month. At an exchange rate of 540 colones per USD, this amounts to $652.15 United States dollars (USD) per month [5]. The annual average income for Costa Rican workers is estimated to be 9,445,151 colones which are equal to $8,745 USD ($728.79 monthly USD) [6].

The latest data released by the CCSS establishes a prevalence of type 2 diabetes of 14.8% in adults over the age of 19 [3]. The International Diabetes Federation Atlas, 2021 edition, reports a prevalence of 10% (95% confidence interval 9.1-17.3%), with diabetes-related expenditure of 2,890 USD per person [7].

Despite diabetes not being listed as one of the top 10 causes of death in the period spanning 2009 to 2019, it is a prevalent risk factor in the two main causes: ischemic heart failure and cerebrovascular disease. Additionally, diabetes was listed as the second major cause of death and disability, alongside the third highest: high fasting glucose blood levels [8].

Overview of the public health system

All workers in Costa Rica must be enrolled in the CCSS. This health cover also grants access to the worker's spouse, children, and parents. All pregnant women and people under the age of 18 are also granted coverage. It is estimated that between the years 2018 and 2021, 91% of Costa Rica’s population was covered and had access to CCSS services [8].

At the CCSS, current diabetes medications include human regular insulin, neutral protamine Hagedorn (NPH) human insulin, glyburide, gliclazide, and metformin. In special situations such as small children, the presence of hypoglycemia that is difficult to manage, or insulin allergies, the health system will provide insulin glargine U100 and insulin lispro. Also, on a case-by-case analysis, some patients may have other anti-diabetic drugs if the treating physician can justify why the patient should receive them instead of the other options available. Since most of the patients are being treated at the primary care level, they will receive only the basic medications. Medications that are used to treat diabetes-associated co-morbidities such as hypertension and dyslipidemia are available, including angiotensin-converting enzyme inhibitors (enalapril), angiotensin II receptor blockers (irbesartan) and statins (lovastatin).

Limitations in the Management at the Primary Care Level

Most of the patients are currently being treated by primary care physicians. The CCSS treatment guidelines [9] establish a treatment goal of glycated hemoglobin (HbA1c) of less than 7%. Its current treatment algorithm starts with metformin, then sulfonylurea (gliclazide is limited to patients over 58 years), and finally insulin therapy. If the patient does not achieve treatment goals, they can be referred to a second level of attention, usually a family physician or internal medicine specialist. Only if the patient has been first evaluated at the second level can they then be referred to a tertiary care center, usually endocrinology or internal medicine. Therefore, it is not surprising that most patients are being treated by the primary care physician, and consequently the path to an evaluation by a specialist is both lengthy and slow, with referred appointments often being months away from the time of referral. Once the patient has been evaluated at the secondary or tertiary care level, they can be referred back to primary care physicians for the continuation of treatment. If a specific complication is detected, patients may be referred directly to a different specialist such as an ophthalmologist, nephrologist, or cardiologist, although specific requirements must be met for some of these specialties (such as radiologic studies, prior use of some medications, prior evaluation at a second level care, etc.).

Alternately, physicians in primary care will have access to most of the required tests, including HbA1c, lipid profile, creatinine, and microalbuminuria. Patients can also be referred directly to an ophthalmologic evaluation by primary care physicians. Patients are required to have their Hba1c analyzed at least every six months, renal function and lipid profile at least once a year, and theoretically, ophthalmologic evaluation once a year. However, the ophthalmologic evaluation presents the previously mentioned limitation of lengthy waiting lists.

Patients can be referred to nutritional counseling by their primary care physician. Consequently, the number of nutritionists is limited, and very often the same issue regarding waiting lists is present as well.

Officially, there is no diabetes educator position as a job post in the CCSS. There are some nurses that have been educated in diabetes and serve as diabetes educators in some hospitals and clinics, but they are few and far between. Furthermore, some primary care physicians may get additional training in diabetes, and work as the coordinator of some clinics for patients with diabetes, although this is not a nationwide established initiative.

Preventive Services

There are no clear public policies regarding the prevention of non-communicable diseases. The prevalence of obesity in Costa Rica is similar to those of developed countries and follows the same increasing trends. Although there are some initiatives to promote exercise and healthy lifestyles, these efforts are not extensive nor effective. At the CCSS, there are no exercise coaches; there are some clinics where patients may go and exercise once a week, but they are not readily available. Nutritional counseling is not accessible, given the number of nutritionists is limited and waitlists are extensive. Most physicians in Costa Rica are ill-equipped to provide specific nutritional counseling. At the CCSS, there are no medications available to treat obesity specifically.

At the government level, there are some initiatives to change the current food labels. However, this is still not mandatory by law. There were some changes at public schools, where they started trying to ban some foods. However, this was not widely accepted and met with some amount of backlash by the system.

Lack of Data

Electronic records have been implemented gradually over the last few years at the CCSS. Despite this, clear data regarding glucose control in patients with type 2 diabetes in the Costa Rican public health system is not available. There have been some reports, however, they’re mostly from the private sector. Additionally, we don’t have data regarding the prevalence of different kinds of complications, either microvascular or macrovascular. Considering the risk of hypoglycemia due to the medications available at the CCSS (human insulins and sulphonylureas), there is no clear data available for the number of consultations in emergency rooms or outpatient services of such episodes.

Screening Services

Adults that go to the primary care physician should have a fasting blood glucose reading performed at least once a year. However, this is not the standard practice for most people, contrary to most of the developed world. Patients will usually go to the physician because they are sick, rather than for preventive reasons.

Once the patients present diabetes, according to current treatment guidelines provided by the CCSS, HbA1c should be performed at least twice a year, lipid profile and renal function once per year, as well as an ophthalmologic evaluation in the same time span. Fasting plasma glucose must be performed in each visit (every three months), however, there is no specific recommendation regarding post-prandial plasma glucose.

Private health system

Physicians are free to have private practice and patients may choose to be treated there. Patients are free to choose which physician and specialty they want to go to. There is also a growing number of patients who have access to private insurance, either through their job or due to the patients acquiring their own additional insurance. If the patient does not have private insurance, all costs acquired through the private health system will be paid out of pocket by the patient. Patients at private centers may have access to some CCSS medications and laboratory tests, through a “mixed medicine” scheme, if they also have public coverage, which in turn lowers the patient’s expenses.

Available Medications

In the private health system, the choice of anti-diabetic drugs is much wider. There are different formulations of metformin, either as monotherapy or in fixed-dose combinations. Different therapeutic classes are available, including DPP-4 inhibitors (sitagliptin, vildagliptin, linagliptin, and saxagliptin), SGLT2 inhibitors (empagliflozin, dapagliflozin, and canagliflozin), sulfonylureas (glyburide, glimepiride, and gliclazide MR), GLP-1 receptor agonists (dulaglutide, semaglutide, and liraglutide), a co-formulation of insulin glargine with lixisenatide, insulin analogs (detemir, glargine U100, glargine U300, degludec, lispro, aspart and glulisine), and premixed insulins (25/75 or 30/70). Also, sensor-augmented continuous subcutaneous insulin infusions are also options. Therefore, in private practice, there is a greater range of options to treat diabetic patients.

Cost of the Medications

The main limitation regarding access to private care and its medications is cost. Even though the cost of these medications is lower compared to developed countries, given our mean income is lower, it represents a larger burden proportionally. Even though generics are available for some older medications, the cost of said pharmaceuticals in Costa Rica is usually only 30-40% lower compared to brand-name drugs.

Table 1 shows the average monthly cost of the full dose of different anti-diabetic drugs in the private market, and how much it represents when compared to Costa Rica’s minimum wage.

**Table 1:** Average private costs of antidiabetic medications in proportion to Costa Rica's minimum wage * Data obtained as an average of two online Costa Rican drugstores (Fischelenlinea.com and farmavalue.com). Accessed on May 1st, 2023. The exchange rate applied is 540 colones per 1 USD. + Minimum wage calculated as 11738.83 colones daily *30 days. The exchange rate is 540 colones per USD.

Drugs	Monthly cost in USD*	Percentage of minimum wage+
Dapagliflozin 10 mg	64.57	9.9%
Empaglifozin 25 mg	54.86	8.41%
Canagliflozin 300 mg	66.68	10.22%
Liraglutide 1.8 mg daily	308.52	47.30%
Dulaglutide 1.5 mg weekly	149.40	22.90%
Semaglutide 1.0 mg weekly	298.61	45.78%
Sitagliptin 100 mg (brand)	61.14	9.37%
Sitagliptin generic 100 mg (Raven)	25.62	3.92%
Linagliptin 5 mg (brand)	50.92	7.80%
Generic linagliptin 5 mg (Square)	32.35	4.96%
Vildaglitin 50 mg bid	54.28	8.32%
Gliclazida MR 60 mg	28.74	4.40%
Glimepiride 4 mg (brand)	68.74	10.54%
Glyburide 5 mg (generic)	5.93	0.91%
Metformin XR 2000 mg daily	72.30	11.08%
Generic metformin 2000 mg daily (Denk)	19.54	2.99%
Insulin glargine U100 40 u/d (brand)	160.98	24.68%
Biosimilar insulin glargine U100 40 u/d (Eli Lilly)	84.90	13.01%
insulin glargine U300 40 u/d	182.58	27.99%
Insulin degludec 40 u/d	157.83	24.20%

Out-of-Pocket Expenditure

The World Bank reported that in 2020 Costa Rica spent 7.86% of its gross domestic product on healthcare [10]. Out of the said amount, 20.29% was out of pocket by its citizens [11]. There is no specific data regarding the out-of-pocket expenditure for the treatment of diabetes mellitus and its complications.

Private Insurance

There are some patients that have private insurance that will cover the attention of patients with diabetes. However, only a small portion of patients have these insurances, either because the patient acquired it or because it is part of some job benefits. Coverage of private insurance will vary, usually requiring a co-payment of around 20% for medications and medical attention. Another difference with developed countries is that, usually, the patient has to pay first for the medical service and medications, file a claim to the insurance company, and then get reimbursed; thus adding a financial burden to patients. Most private insurance will cover the different kinds of anti-diabetic drugs. However, lately, access to GLP-1 receptor agonists (specially semaglutide) is being restricted to those patients that have failed to respond to other kinds of drugs, and in some cases, a pre-authorization for its use has to be issued first.

Analysis

Costa Rica has a robust public health system, where clinical care is provided by the CCSS. On the bright side, its ample coverage of more than 90% of Costa Rica’s population allows most of its inhabitants to have access to consistent diabetes treatment. However, its treatment is limited by the number of medications that are available, a referral system that is lengthy, and under-provided access to support services such as nutritional and exercise counseling. This is worsened by the fact that there are no diabetes educators in the system. Additionally, the health system’s performance regarding glucose control is unknown. Prevalence, prevention, and treatment of complications and co-morbidities are also incognito in the system. Therefore, it is difficult to assess how effective the treatment of diabetes is. Despite the surge of new therapeutic options for the treatment of diabetes in the last decades, none of these are readily available at the CCSS. Newer medications with evidence of cardio-renal protection (such as SGLT2 inhibitors and GLP1 receptor agonists) are not available to patients. Extensive use of these medications may not only contribute to glucose control but also decrease the burden of complications.

As far as preventive measures go, despite the increasing prevalence of overweight and obesity, the CCSS lacks a clear strategy to prevent diabetes and its complications. Access to nutritional counseling and physical activity educators is cumbersome and mostly unavailable to primary care patients.

Private health care is very easy to access (waitlists are minimal to nonexistent) and most anti-diabetic drugs are available for prescription in the private market. For those with private insurance or sufficient economic resources, it allows them to have access to a very ample spectrum of treatment possibilities, similar to those in developed countries. However, its main limitation is precisely cost. Given the financial burden represented by these out-of-pocket expenses, it is often unrealistic for most Costa Ricans (in proportion to their wages).

Ultimately, we have two different sides of Costa Rica. On the one hand, for those who have access to the private health system, treatment of diabetes mellitus should not be very different from the worldwide standards in 2023. On the other hand, for those in the public health system, the choices are limited and they do not have access to newer anti-diabetic drugs.

Recommendations

Given the previous discussion, it is imperative that we pose the following question: how can we improve the care of patients with diabetes in Costa Rica? Firstly, from a preventative point of view, there is a need for a clear public health policy where there is more access to safe public spaces to exercise, combined with nutritional guidance. The public health system should facilitate access to both nutritional and exercise counseling. Secondly, there should be greater access to new anti-diabetic drugs that are already included in the list of essential medicines by the World Health Organization, such as SGLT2 inhibitors and insulin analogs. These drugs may improve glucose control, as well as provide organ protection with fewer side effects, such as hypoglycemia. Thirdly, the inclusion of new anti-diabetic drugs must be accompanied with no exception by medical education programs, where primary care physicians learn how to treat patients with diabetes and how to use these newer medications. Greater effort should be directed toward creating the diabetes educator job post, and all patients with diabetes should have access to these educators, as they may facilitate nutritional and exercise counseling.

We should also have better data regarding the control of diabetes and the prevalence of its complications. This data should be easily available through the electronic medical records of the public health system and may guide future public policies and the CCSS’s type 2 diabetes guidelines.

In the private sector, most of the patients who do not have private insurance, healthcare, and drug expenditure are out of pocket. Therefore, medication costs can be lowered by promoting the use and availability of good quality generic drugs at an affordable price. Private insurance should reimburse all medications that are used to treat diabetes, and GLP1 receptor agonists should be no exception.

Conclusions

Costa Rica’s public health system has an extensive coverage of more than 90% of the total population. Most patients are being treated at the primary care level, and access to anti-diabetic drugs is limited to sulphonylureas, metformin, and human insulins. Referral to specialists (such as internal medicine or endocrinology) and nutritional counseling are available although usually with long waitlists. In the private sector, newer anti-diabetic drugs are available but most of the expense is out of pocket and limits the access to these drugs. Some patients with private insurance will have some kind of reimbursement for private medical attention and prescription of anti-diabetic drugs, however, this does not cover the large majority of patients.
